# Phenotypic and genetic markers of psychopathology in a population-based sample of older adults

**DOI:** 10.1038/s41398-021-01354-2

**Published:** 2021-04-24

**Authors:** Arianna M. Gard, Erin B. Ware, Luke W. Hyde, Lauren L. Schmitz, Jessica Faul, Colter Mitchell

**Affiliations:** 1 Department of Psychology, University of Maryland, College Park, MD USA; 2 Institute for Social Research, University of Michigan, Ann Arbor, MI USA; 3 Department of Psychology, University of Michigan, Ann Arbor, MI USA; 4 La Follette School of Public Affairs, University of Wisconsin, Madison, WI USA

**Keywords:** Genomics, Psychiatric disorders

## Abstract

Although psychiatric phenotypes are hypothesized to organize into a two-factor internalizing–externalizing structure, few studies have evaluated the structure of psychopathology in older adults, nor explored whether genome-wide polygenic scores (PGSs) are associated with psychopathology in a domain-specific manner. We used data from 6003 individuals of European ancestry from the Health and Retirement Study, a large population-based sample of older adults in the United States. Confirmatory factor analyses were applied to validated measures of psychopathology and PGSs were derived from well-powered genome-wide association studies (GWAS). Genomic SEM was implemented to construct latent PGSs for internalizing, externalizing, and general psychopathology. Phenotypically, the data were best characterized by a single general factor of psychopathology, a factor structure that was replicated across genders and age groups. Although externalizing PGSs (cannabis use, antisocial behavior, alcohol dependence, attention deficit hyperactivity disorder) were not associated with any phenotypes, PGSs for major depressive disorder, neuroticism, and anxiety disorders were associated with both internalizing and externalizing phenotypes. Moreover, the variance explained in the general factor of psychopathology increased by twofold (from 1% to 2%) using the latent internalizing or latent one-factor PGSs, derived using weights from Genomic Structural Equation Modeling (SEM), compared with any of the individual PGSs. Collectively, results suggest that genetic risk factors for and phenotypic markers of psychiatric disorders are transdiagnostic in older adults of European ancestry. Alternative explanations are discussed, including methodological limitations of GWAS and phenotypic measurement of psychiatric outcome in large-scale population-based studies.

## Introduction

Psychiatric disorders impact health, wealth, and wellbeing across the life course^1–3^. In the United States, common psychiatric disorders such as major depressive disorder (MDD) are among the top 10 leading causes of disability and injury^4^. Among older adults, psychiatric disorders have pronounced effects on physical health and mortality^2,5^. Moreover, the 12-month prevalence of having any psychiatric disorder in older adulthood is substantial, with recent estimates of 11.5%^6^. As the number of Americans older than 65 years is projected to double in the coming decades^7^, more research is needed to understand the presentation and etiology of psychiatric illness in older adults.

### Phenotypic structure of psychiatric disorders

Psychiatric disorders show marked comorbidity across developmental stages^8,9^. A robust literature suggests that this comorbidity may be explained by an overarching phenotypic meta-structure that includes separate but correlated internalizing (e.g., depression, anxiety) and externalizing (e.g., substance use, attention deficit hyperactivity disorder [ADHD]) factors^10^. These comorbidity patterns align with phenotypic differences between internalizing disorders, characterized by elevations in negative affect^11^, and externalizing disorders, characterized by behavioral disinhibition^12^. Alternatively, a single factor (or a bifactor) model that explains shared variance across all psychiatric disorders has also been supported^10,13^, and may emerge in developmental stages where symptoms are less prevalent (e.g., early childhood, older adulthood)^14^. Yet examinations of the meta-structure of psychiatric comorbidity have focused primarily on child and younger adult samples. The lack of attention to older adults is a striking omission given the still substantial and impairing rates of psychiatric disorders in this population^2,5,6^. Moreover, given clear gender (i.e., greater internalizing symptoms among women, and greater externalizing symptoms among men) and age (i.e., decreasing prevalence of psychiatric disorders across both domains) differences in the prevalence of psychiatric disorders^6,15,16^, more research is needed to determine how the meta-structure of psychopathology varies across these demographic groups in older adults.

### Genetic architecture of psychiatric disorders

Genetic risk for psychiatric disorders may also align in a two-factor meta-structure. Twin and family designs suggest that additive genetic risk accounts for the two-factor internalizing–externalizing meta-structure^16,17^, and data from genome-wide association studies (GWAS) has been leveraged to identify single-nucleotide polymorphisms (SNPs) that are unique to internalizing or externalizing disorders^18^. Yet, there is also evidence of shared genetic risk across internalizing and externalizing domains^14,19–21^, including data from a psychiatric cross-disorder GWAS meta-analysis showing that genetic risk variants are enriched for biological processes core to many psychiatric conditions^21^.

As psychiatric disorders are highly polygenic (i.e., resulting from both common variants of small effect, likely to impact many psychiatric disorders, and rare variants of larger effect, possibly unique to certain phenotypes)^22,23^, polygenic score (PGS) estimation is one tool that can be used to capture psychiatric polygenicity. A PGS is constructed as sum score of risk alleles that an individual has, weighted by the risk allele effect size from a GWAS in an independent sample^24^. Although PGSs are constructed for a specific phenotype (e.g., MDD), PGS analyses have revealed widespread cross-phenotype correlations^25^. For example, a phenome-wide analysis in young adults indicated that a PGS of depressive symptoms was associated with several phobias and generalized anxiety disorder but not externalizing phenotypes, whereas a PGS for smoking initiation was associated with antisocial behavior but not internalizing phenotypes^26^. For researchers studying the etiology of psychiatric illnesses, such widespread associations present a methodological challenge: which PGS best captures genetic risk for a single psychiatric disorder? Given this issue of overlapping genetic risk, quantitative approaches to combining PGSs are needed. Recent methodological innovations have enabled users to construct better-performing PGSs by taking advantage of cross-trait correlations^27–29^. However, such cross-trait “latent” PGSs have only been applied to cohorts aggregated across age groups^21^. In large, population-based samples of older adults, the relative performance of individual PGSs and latent PGSs for specific psychiatric outcomes and general psychopathology is yet unknown.

### Current study

We assessed the meta-structure of psychopathology in a large population-based sample of 6003 older adults from the Health and Retirement Study (HRS). We examined whether two-factor phenotypic models fit the data better than one-factor models and further probed the invariance of these models across gender and age. Second, we examined whether there was polygenic specificity in the associations between PGSs for psychiatric (e.g., MDD, ADHD) outcomes and behaviors indexing psychopathology (e.g., cannabis use, antisocial behavior). Next, we implemented Genomic SEM to derive latent PGSs based on the genetic architecture of GWAS summary statistics and present the first analyses of how these latent cross-trait PGSs perform in a large population-based sample of older adults. Based on research in younger samples, we hypothesized that a two-factor model would fit the phenotypic data better than a one-factor model and that PGS-phenotype associations would be hierarchically organized, such that PGSs for internalizing disorders would be more strongly associated with internalizing outcomes, and PGSs for externalizing disorders would be more strongly associated with externalizing outcomes.

## Methods and materials

### Sample

Data were drawn from the HRS, a nationally representative longitudinal panel study of over 43,000 adults over age 50 and their spouses^30^. Launched in 1992, the HRS introduces a new cohort of participants every 6 years and interviews ~20,000 participants every 2 years. Eligible participants for the current study (*N* = 6003; 58.0% female; mean age in years [SD] = 67.49 [8.14]) were of genetically European ancestry (i.e., because PGSs were constructed from European Ancestry GWAS) and participated in the Leave-Behind Psychosocial Questionnaire^31^ in 2010 or 2012. Participants younger than 51 years were excluded because they were not part of the original sampling frame, as were participants who completed the Leave-Behind Psychosocial Questionnaire in institutional settings, and participants who were born before 1930 (i.e., to address concerns for selective mortality^32^). Within the analytic sample, 52% earned a high school diploma and 27.4% earned a four-year college degree or higher. Beyond the exclusion criteria listed above, compared to the total HRS, the analytic sample had a higher proportion of women (*χ*²(1) = 11.085, *p* < 0.001), but did not differ on years of schooling (*t*[38181] = 1.79, *p* > 0.05). Informed consent was obtained for all participants, and study procedures were approved by the Institutional Review Board at the University of Michigan. All HRS phenotypic data and PGSs are publicly available at https://hrs.isr.umich.edu/data-products. Quality-controlled genetic data are available on dbGaP (https://www.ncbi.nlm.nih.gov/gap/).

### Phenotypic measures

Although the HRS was not explicitly designed to study psychiatric outcomes, several available measures capture dimensional symptoms of psychopathology and related traits. Measures were drawn from the Leave-Behind Psychosocial Questionnaire, a self-reported questionnaire administered to a random 50% of the core HRS participants at each biennial wave during face-to-face interviews^31^. A complete wave of data was constructed using the 2010 and 2012 data collections. Depressive symptomatology and drinking frequency were taken from RAND HRS 2010 and 2012 Fat Files^33^. All phenotypic data are publicly available through the HRS website (http://hrsonline.isr.umich.edu/).

Measures of internalizing psychopathology included negative affect^34^, anxiety symptoms^35^, and depressive symptoms^36^; externalizing psychopathology was captured by impulsivity^37^, trait and state anger^38^, and the number of drinks per day (Supplemental Table 1). Although some of these measures (e.g., impulsivity) do not capture psychopathology per se, the constructs are consistent with a dimensional model of psychopathology (e.g., Hierarchical Taxonomy of Psychopathology (HiTOP)) and the Research Domain Criteria (RDoC) framework^39,40^. See Supplemental Table 1 for details.

### Genetic data and PGSs

A random subset of the ~26,000 total participants was selected to participate in enhanced face-to-face interviews and saliva specimen collection (for DNA) in 2006, 2008, 2010, and 2012. Genotyping was conducted by the Center for Inherited Disease Research (CIDR) in 2011, 2012, and 2015. Genotype data on over 15,000 HRS participants was obtained using the llumina HumanOmni2.5 BeadChips (HumanOmni2.5-4v1, HumanOmni2.5-8v1, and HumanOmni2.5-9v1.1), which measures ~2.4 million SNPs. Individuals with missing call rates >2%, SNPs with call rates <98%, HWE *p* value <0.0001, chromosomal anomalies, and first-degree relatives in the HRS were removed. The current paper uses data from unrelated HRS participants of European genetic ancestry (*n* = 9991) from the genetic data collection years of 2006, 2008, and 2010. Genetic ancestry was determined in a two-stage PCA process wherein the final European American sample included all self-reported non-Hispanic whites that had PC loadings within ±1 SD of the mean for eigenvectors 1 and 2 in the PC analysis of all unrelated study subjects. PCA was then used again within the European American sample to estimate the top 10 “ancestry-specific” PCs (see Supplemental Methods for more detail).

PGSs of internalizing (neuroticism^41^, any anxiety disorder^42^, MDD^43^) and externalizing (alcohol dependence^44^, ADHDH^45^, cannabis use^46^, and antisocial behavior^47^) psychopathology were constructed using well-powered, European ancestry GWAS summary statistics (Table 1). If the original GWAS included the HRS, we obtained summary statistics with the HRS sample removed (for more detail, see https://hrs.isr.umich.edu/data-products/genetic-data;^48^). Although GWAS summary statistics are available for other psychiatric disorders (e.g., schizophrenia, bipolar disorder), we did not construct these PGSs because these phenotypes were not measured in the HRS. A PGS for height was included as a negative control^49^. To construct PGSs, SNPs in the HRS genetic data were matched to SNPs with reported results in each GWAS (see Table 1 for the number of SNPs that contributed to each PGS). As we only used genotyped SNPs (i.e., no imputation) to construct PGSs, we did not trim based on linkage disequilibrium, nor did we impose a GWAS *p* value threshold/cutoff for included SNPs^48^. The PGSs were calculated as weighted sums of the number of phenotype-associated alleles (zero, one, or two) at each SNP, multiplied by the effect size for that SNP estimated from the GWAS meta-analysis. All SNPs were coded to be associated with increasing disease risk. To simplify interpretation, the PGSs were normalized within the European ancestry sample. All analyses in which PGSs were combined with phenotypes included the top 10 ancestry-specific genetic principal components as covariates.

**Table 1:** GWAS summary statistics used to construct polygenic scores.

Construct	GWAS discovery *N*	*N*_overlapping SNPs_ with HRS genetic data	Citation	Location of GWAS summary statistics
Neuroticism	170,911	1,134,281	Okbay et al. (2016)	https://www.thessgac.org/data
Anxiety	7,016 cases/14,745 controls	1,079,599	Otowa et al. (2016)	https://www.med.unc.edu/pgc/results-and-downloads
Major depressive disorder	59,851 cases/113,154 controls*	1,340,536	Wray et al. (2018)	https://www.med.unc.edu/pgc/results-and-downloads
Alcohol dependence	11,569 cases/34,999 controls	1,170,144	Walters et al., (2018)	http://www.med.unc.edu/pgc/results-and-downloads
Attention deficit/ hyperactivity disorder	20,183 cases/35,191 controls	1,043,408	Demontis et al. (2017)	http://www.med.unc.edu/pgc/results-and-downloads
Cannabis use	162,082	1,310,508	Pasman et al., (2019)	https://www.ru.nl/bsi/research/group-pages/substance-use-addiction-food-saf/vm-saf/genetics/international-cannabis-consortium-icc/
Antisocial behavior (continuous)	16,400	1,289,915	Tielbeek et al. (2017)	http://broadabc.ctglab.nl/summary_statistics

^*^The MDD GWAS weights used to construct polygenic scores were based on publicly available data only and thus, did not include 23andMe data, due to data use agreements. GWAS summary statistics were obtained without the Health and Retirement Study as a contributing sample.

#### Genomic SEM

To complement our analyses using individual PGSs within the HRS, we implemented Genomic Structural Equation Model (SEM) to construct latent PGSs^28^. Genomic SEM models the genetic covariance structure of GWAS summary statistics and allows for model comparison of different confirmatory factor models (e.g., one factor versus two factor). SNPs can be integrated into the modeling framework to estimate new SNP effects on cross-trait genetic liability, thus allowing for the generation of new PGSs for latent traits. Using the same GWAS summary statistics used to construct PGSs in the HRS, we estimated and compared one-factor and two-factor models of genetic risk for psychopathology. Following Genomic SEM, we constructed models of latent PGSs within the HRS, using the same methods described above. It is important to note that traditional confirmatory factor analyses could not be used to evaluate the structure of PGSs because many of the original GWAS included the same participants. Although LD-score regression^20^ can be used to determine cryptic relatedness by evaluating the cross-trait LD-score regression intercepts, our analyses revealed substantial sample overlap (Supplemental Fig. 1). By contrast, Genomic SEM produces model parameters and test statistics that are unbiased by patterns of shared estimate error across the original GWASs^28^.

### Analytic strategy

Analytic code for the current paper is available at https://osf.io/c9uj8/. All analyses and visualizations were conducted in R Statistical Software^50^. To increase generalizability and avoid overfitting the data, the analytic sample (*N* = 6003) was divided into two random samples of *n* = 3002 and *n* = 3001. One data set (i.e., the “test sample”) was used to estimate phenotypic one-factor and two-factor models using confirmatory factor analyses; the second data set (i.e., “the hold-out sample”) was used to replicate the best-fitting factor structure. Confirmatory factor analysis is a theory-driven form of structural equation modeling that can be used to capture the shared variance among observed correlated variables to estimate unobserved latent factors^51^. The model fitting procedure compares the model implied covariance matrix to the observed covariance matrix, allowing users to compare model fit using several indices. We considered model fit acceptable if the root mean square error of approximation (RMSEA) < 0.06, and the Comparative Fit Index (CFI) and Tucker Lewis Index (TLI) >0.90^52^. One-factor and two-factor models were compared using ∆CFI and ∆RMSEA as alternatives to chi-square difference testing, which is sensitive to large sample sizes^53^; a ∆CFI > −0.01 and ∆RMSEA > 0.015 indicates significant depreciation of model fit^54,55^. All models were estimated using maximum likelihood estimation with robust standard errors in the *lavaan* package^56^. Maximum likelihood estimation can be used to account for missing data (in the current study, there was <5% missing phenotypic data and no missing genetic data) and outperforms other approaches to missing data such as listwise deletion and multiple imputation^57^.

The semTools package^58^ was used to estimate measurement invariance across gender (1 = male, 2 = female) and age. To examine invariance across age, we split the sample into three groups: middle age (51–64 years), young–old (65–74), and old–old (75–83). Previous research has documented developmental differences by these age groupings, including environmental effects on depressive symptoms^59^, self-rated health^60^, and familial social support^61^. Increasingly stringent models of invariance across groups are tested: (a) configural invariance—same underlying structure with all parameters freely estimated across groups, (b) metric invariance—invariant loadings across groups, (c) scalar invariance—invariant factor loadings and intercepts across groups, and (d) residual invariance—invariant factor loadings, intercepts, and unique factor variances across groups^62^.

Linear regression was used to examine the effects of the individual PGSs and the latent PGSs (estimated using GWAS summary statistics within Genomic SEM) on latent phenotypic factors, controlling for the top 10 ancestry principal components. These analyses were conducted within the hold-out sample only (*n* = 3001). In large sample sizes, most estimates will be significant at the 95% confidence level. Therefore, we used G*Power^63^ to estimate expected effect sizes; assuming 80% statistical power, an alpha error probability of 0.05, and a sample size of *N* = 3003, we are statistically powered to interpret models with an adjusted *R*^2^ ≥ 0.008.

## Results

**Fig. 1:**
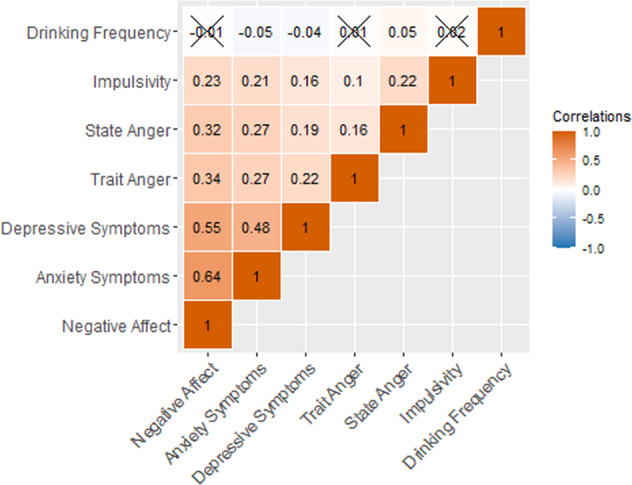
Within- and across-domain correlations among phenotypes in the Health and Retirement Study. 5873 < *N* < 5965. Associations that were not significant at *p* < 0.05 are marked with an “X”.

Correlations revealed greater within-domain associations among internalizing phenotypes (0.48 < *r* < 0.64) than externalizing phenotypes (0.05 < *r* < 0.22; Fig. 1). However, there were also significant positive cross-domain associations (0.16 < *r* < 0.34). For example, depressive symptoms were positively associated with all the externalizing phenotypes except drinking frequency (Fig. 1). We used the *effectsize* package^64^ to compare the effect sizes of the correlations among and between HRS phenotypes to the effect sizes reported in a previous study that relied on structured clinical interviews in a sample of older adults^9^. Most associations observed in the HRS were similar in effect size; the only effects that were substantially weaker in the HRS were the associations between drinking frequency and the other externalizing and internalizing measures. Of note, drinking frequency was the only measure for which reliability could not be estimated; all other HRS measures have Cronbach’s alphas of 0.96–0.97 (Supplemental Table 1).

### Phenotypic models

**Fig. 2:**
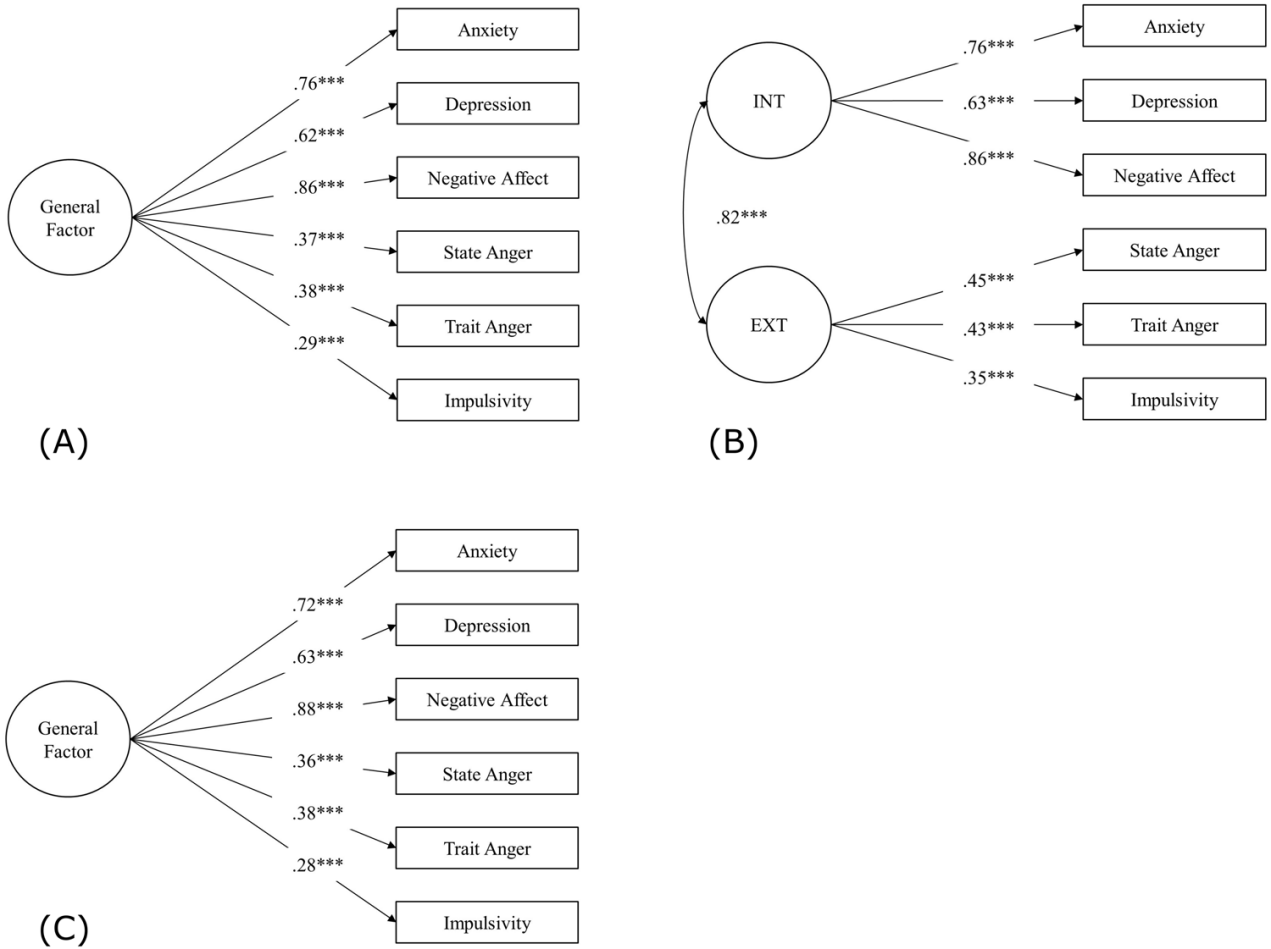
High correlation between internalizing and externalizing factors suggests a one-factor model of psychopathology among older adults in the Health and Retirement Study. *INT* internalizing, *EXT* externalizing. Standardized estimates are shown. **A**, **B** Confirmatory one-factor (model fit: *χ*²(9) = 70.37, *p* < 0.001; CFI = 0.980; TLI = 0.967; RMSEA = 0.052, 90% CI [0.041, 0.064]) and two-factor (model fit: *χ*²(8) = 46.71, *p* < 0.001; CFI = 0.988; TLI = 0.977; RMSEA = 0.044, 90% CI [0.032, 0.056]) phenotypic models in the test sample (*n* = 3002). **C** Confirmatory one-factor phenotypic model in the hold-out sample (*n* = 3001; model fit: *χ*²(9) = 61.96, *p* < 0.001; CFI = 0.983; TLI = 0.972; RMSEA = 0.048, 90% CI [0.037, 0.059]).

Next, we evaluated one-factor and two-factor phenotypic models. In the test sample (*n* = 3002), drinking frequency loaded negatively on the latent factor(s) and was dropped from subsequent analyses (results available upon request). Figure 2A, B displays the one-factor and two-factor phenotypic models in the test sample (*n* = 3002), which both fit the data well. Although the relative fit indices suggested that the two-factor model fit the data better than the one-factor model (i.e., larger CFI and TLI, smaller RMSEA), the ΔCFI and ΔRMSEA were smaller than suggested values^54,55^, indicative of equivalent model fit. The association between the internalizing and externalizing latent factors in the two-factor model was very large (*r* = 0.82), whereas previous work in younger samples reports that the cross-domain correlation hovers ~0.50^10^. These results suggest that the internalizing and externalizing factors do not represent unique constructs in this sample of older adults in the HRS. Thus, we accepted the one-factor phenotypic model in the test sample. Figure 2C shows the one-factor model in the hold-out sample (*n* = 3001). The largest loadings for the general factor of psychopathology were negative affect (*β* = 0.88, *p* < 0.001) and trait anger (*β* = 0.38, *p* < 0.001). The general factor explained far more variance in the internalizing indicators (0.40 < *R*^2^ < 0.78) than the externalizing indicators (0.08 < *R*^2^ < 0.15).

We found evidence for metric invariance of the one-factor model of general psychopathology by gender and age group: fixing the indicator loadings to be equivalent across groups did not significantly degrade model fit (see Supplemental Fig. 2). As expected, given significant mean-level gender- and age-differences in internalizing and externalizing behaviors (see Supplemental Results), models did not meet criteria for scalar measurement invariance (i.e., equivalent intercepts across groups).

### PGS associations with psychopathology

**Fig. 3:**
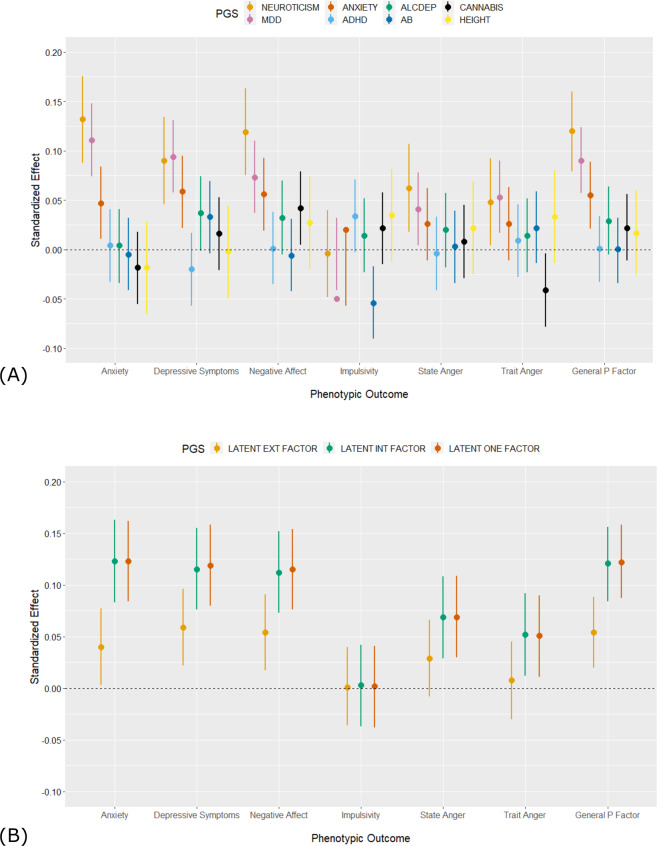
Polygenic scores for internalizing, but not externalizing, disorders are associated with internalizing and externalizing behaviors in the Health and Retirement Study. *N* = 3001. Associations between polygenic scores (PGS) and phenotypic outcomes, accounting for the top 10 ancestry principal components. Estimates are unstandardized and error bars are standard errors. **A** Individual PGSs as predictors; **B** Latent PGSs, where SNP weights were estimated using Genomic SEM. In both panels, error bars are standard errors around the estimate.

To address a critical issue in the field, we evaluated polygenic specificity by examining the associations between each PGS and each phenotypic measure, controlling for the first 10 ancestry-specific principal components. Across all phenotypic outcomes, the predictive power of the externalizing PGSs was low in the HRS sample (Fig. 3A). The only significant association between an externalizing PGS and a phenotypic outcome was a negative association between the PGS for antisocial behavior and impulsivity in older HRS participants. By contrast, the PGSs for neuroticism, MDD, and anxiety were significantly positively associated with anxiety, depressive symptoms, negative affect, and the general latent factor of psychopathology (*R*^2^ values ~1%). The PGS for height was not associated with any phenotypic measures.

### Genomic SEM and latent PGSs for psychopathology

**Fig. 4:**
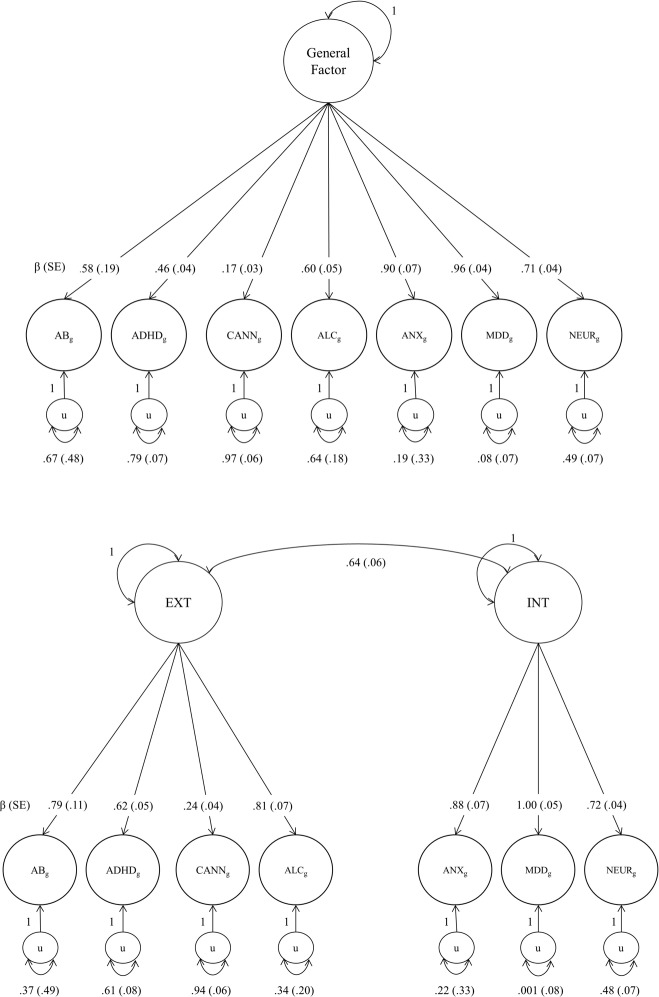
Genomic SEM one-factor and two-factor model. Confirmatory factor analyses were conducted on the GWAS summary statistics in Table 1, using the Genomic SEM package in R Statistical Software (Grotzinger et al., 2019). Standardized estimates are shown. See Supplemental Fig. 3 for unstandardized estimates. In both the one-factor and two-factor models, the residual variance of MDD was fixed to zero. Model fit comparisons between the one-factor model (*χ*²(14) = 76.762, *p* < 0.001, AIC = 104.762, CFI = 0.962, SRMR = 0.127) and two-factor model (*χ*²(13) = 46.072, *p* < 0.001, AIC = 76.072, CFI = 0.980, SRMR = 0.084) indicated superior model fit of the two-factor model (Δ*χ*² = 30.69(1), *p* < 0.001, ΔCFI > 0.01, lower AIC). Single-nucleotide polymorphism effects were then integrated into the model to derive new SNP weights for the construction of latent polygenic scores (see Supplemental Methods).

Genomic SEM was used to fit one-factor and two-factor models of genetic risk for psychopathology, using GWAS summary statistics from well-powered studies of neuroticism^41^, any anxiety disorder^42^, MDD^43^, alcohol dependence^44^, ADHD^45^, cannabis use^46^, and antisocial behavior^47^. Estimated SNP effects were then used to generate PGSs for latent traits in the HRS sample of older adults. Although both the one-factor and two-factor models fit the data well (Fig. 4, Supplemental Fig. 3), model fit comparisons indicated superior model fit of the two-factor model of genetic risk for psychopathology (Δ*χ*² = 30.69^1^, *p* < 0.001, ΔCFI > 0.01, lower Akakie Information Criterion [AIC]). Moreover, the cross-trait correlation was *r* = 0.64, indicating that the internalizing and externalizing latent genetic factors, though correlated, capture different underlying constructs. Owing to small negative residual variance in the two-factor model, the loading for MDD was fixed to 1. The largest loadings on the latent externalizing factor were alcohol dependence (*β* = 0.81) and antisocial behavior (*β* = 0.79). The largest loading on the latent internalizing factor, aside from MDD, was anxiety (*β* = 0.88). As the model fit for the one-factor model was excellent (*χ*²^14^ = 76.762, *p* < 0.001, AIC = 104.762, CFI = 0.962, SRMR = 0.127), we constructed both the latent one-factor PGS and latent internalizing and externalizing PGSs.

Associations between the latent PGSs and phenotypic outcomes indicated that the latent internalizing PGS and latent one-factor PGS explained 1% more variance in the general factor of psychopathology than any of the individual PGSs that were used to construct these latent measures of polygenic risk (i.e., *R*^2^ = 2% versus *R*^2^ = 1%; Fig. 3B). There were no differences in the predictive power of the latent internalizing PGS and the latent one-factor PGS, as indicated by non-overlapping confidence intervals of the standardized effects. Pooling the summary statistics of the externalizing GWAS (i.e., alcohol dependence, cannabis use, ADHD, antisocial behavior) similarly resulted in novel associations with internalizing phenotypes and the general factor of psychopathology, as compared with any of the individual externalizing PGSs. However, the model *R*^2^ was <1% and there were no associations between the latent externalizing PGS and any of the externalizing outcomes.

## Discussion

We evaluated both the phenotypic and polygenic structure of psychopathology in a large population-based sample of older adults. In models that replicated using a split-half design, phenotypes were organized in a one-factor model of psychopathology rather than the two-factor internalizing–externalizing structure more common in younger samples^10,13^. The general factor of psychopathology was further equivalent across gender and age groupings as indicated by invariant factor structure and loadings, suggesting that the structure of psychiatric phenotypes in the HRS is replicable across demographic groups. PGS analyses revealed that genetic risk scores derived from GWAS of externalizing psychopathology are not portable to older adults in the HRS: none of the externalizing PGSs were associated with externalizing or internalizing phenotypes. By contrast, the internalizing PGSs were predictive of internalizing phenotypes and the general factor of psychopathology in the current sample. Perhaps most importantly, using Genomic SEM^29^, we found that the latent internalizing PGS and the latent one-factor PGS explained double the variance than any of the individual PGSs in models predicting internalizing phenotypes and the general factor of psychopathology. Collectively, these results make important contributions to our understanding of transdiagnostic risk for psychopathology—at phenotypic and genetic levels of analysis. For researchers and clinicians interested in the etiology and course of psychopathology in older adults, modeling general psychopathology is likely to improve predictive accuracy and may be important in developing interventions to reduce the burden of mental illness in the second half of the lifespan.

In contrast to research in children and adults^10,13^, psychiatric phenotypes in the HRS sample of older adults organized into one general factor of psychopathology rather than a two-factor internalizing–externalizing factor structure. Identification of the meta-structure of psychiatric phenotypes in older adults has both etiological and clinical implications. First, the largest loading on the general factor was negative affect. Negative affect or negative emotionality is thought to be a non-specific vulnerability factor for multiple forms of psychopathology^65^, is correlated with both internalizing and externalizing disorders^66^, and is oftentimes the first factor extracted from individual differences in dispositional traits^65,67,68^. That negative affect as a dispositional construct is robustly associated with multiple symptom domains^13^ supports the RDoC framework from the National Institute of Mental Health, in which the biological origins of intermediate phenotypes are linked to multiple categorical disorders^40^. Our results further support the HiTOP approach^39^, which advocates for dimensional approaches that better characterize psychiatric comorbidity across symptom domains compared to traditional categorical nosologies. Clinically, interventions designed for one disorder have widespread effects on multiple disorders within the same domain^69^. For example, pharmacological and psychosocial interventions designed to treat depression are also effective in treating some forms of anxiety^70^, which has led to transdiagnostic interventions for emotional disorders broadly^71^.

One major contribution of our results is the lack of specificity in PGS prediction of psychiatric phenotypes. It is surprising that a PGS designed to capture genome-wide genetic risk for a single disorder (e.g., MDD) was no better at predicting within a domain (e.g., depressive symptoms) than cross-domain (e.g., state anger) phenotypes. One explanation for these results is that psychiatric GWAS rarely account for comorbidity (e.g., MDD cases without comorbid substance use disorder). By ignoring psychiatric comorbidity, GWAS may be identifying genetic risk factors for multiple phenotypes or clinical severity instead of a single phenotype. Examples of psychiatric genetic studies that account for comorbidity include a study of bipolar disorder and schizophrenia^72^ and a GWAS of comorbid depression and alcohol dependence^73^. Precision phenotyping of homogenous subgroups (e.g., stratification by age of disorder onset) is also likely to improve to GWAS and resultant PGSs^74,75^.

Using Genomic SEM, polygenic risk organized into a two-factor internalizing–externalizing structure, although the one-factor model also fit the data well. Importantly, these latent PGSs that aggregated genetic effects across multiple GWAS explained 1% more variation in the general factor of psychopathology. As MDD was the largest loading in both the one-factor and two-factor Genomic SEM models (Fig. 4), it may not be surprising that there were no differences in the predictive power of the latent internalizing PGS and the latent one-factor PGS. Collectively, our results reiterate the power of aggregating genetic effects across multiple related phenotypes^29^ and suggest that any researcher interested in capturing genome-wide genetic risk for psychopathology should implement methods to aggregate GWAS summary statistics of similar phenotypes rather than rely on PGSs of individual disorders.

In addition to practical implications, our results demonstrate that genetic risk for psychiatric phenotypes is transdiagnostic. Psychiatric GWAS repeatedly show that associated SNPs tend to cluster in genes underlying neurodevelopmental processes, signal transduction, and synaptic plasticity^21,41,43^, all processes common to complex diseases. Moreover, biometric analyses in behavioral genetic/family designs demonstrate that a general genetic factor influences multiple psychiatric disorders (and their overlap) and explains more of the variation in psychiatric outcomes than the unique internalizing and externalizing genetic effects^19,76^. More research is needed to understand whether psychiatric polygenic risk is pleiotropic and if so, what kind of pleiotropic processes are at play. For example, biological pleiotropy would suggest that a genetic risk variant for neuroticism (or another intermediate transdiagnostic phenotype) predicts multiple disorders^77^. By contrast, mediated pleiotropy would suggest that a genetic risk variant predicts one phenotype (e.g., neuroticism), which subsequently predicts the onset of other phenotypes (e.g., alcohol use). Longitudinal phenotypic data and causal inferences techniques^78^ are needed to evaluate these hypotheses.

A second explanation for low polygenic specificity in the current study is that PGSs are derived from GWAS of common genetic variation—most often SNPs with minor allele frequencies >1%^79^. An “omnigenic model of complex traits” suggests that SNPs that contribute to the bulk of heritability in complex disorders are spread across the genome as common variants of small effect that contribute to cellular processes (e.g., protein binding, sequence-specific DNA binding) relevant to many complex disorders. Disease-specific genetic risk variants, by contrast, are likely to be rare variants of large effect that are often not captured in GWAS of common genetic variation^22,23^. Moreover, GWAS do not capture copy number variants, which are also linked to psychiatric disorders and may function in a disease-specific manner^80^. Thus, it may also be that PGSs derived from GWAS of common genetic variants are not appropriate for examinations of disorder-specific etiology.

Collectively, these results challenge the notion of specificity in the phenotypic and genetic presentation of psychopathology in older adults. The still impairing rates of internalizing and externalizing disorders during the second half of the lifespan necessitate discussion regarding the clinical utility of current diagnostic categories, particularly as we investigate psychiatric etiology using biological approaches such as genetics and neuroscience.

### Limitations

Although the current study is the first to evaluate the meta-structure of phenotypic and genetic risk for psychopathology in older adults using a large, population-based sample, several limitations are worth noting. First, the estimation of latent factors in confirmatory factor analysis is dependent upon the quality of the indicators. Based on previous recommendations^48,81^, we only constructed PGSs based on large GWAS meta-analyses with independent replication samples. As a result, we did not include PGSs derived from smaller GWAS of relevant phenotypes, including several studies of externalizing disorders^82,83^. Relatedly, the phenotypic measures available in the HRS are abbreviated scales, as is common in large surveys. Thus, one alternative phenotypic model that we were unable to fit is a bifactor model of psychiatric outcomes (our models did not converge, likely owing to the sparse measurement of symptoms), which posits that there are internalizing and externalizing factors as well as a higher-order bifactor that captures shared variance between the lower-order factors^10,13,39^; more recent empirical work further suggests that there may be several higher-order bifactors that capture severity in symptoms^84^. Indeed, we observed a high correlation between the internalizing and externalizing factors in the HRS sample, which is thought to indicate the presence of a higher-order bifactor^13,85^. Moreover, the gold standard for measuring psychiatric symptoms and disorders is through structured clinical interviews—e.g., the Structured Clinical Interview for DSM Disorders^86^—or via questionnaires administered to multiple informants^87^. For example, several previous investigations of the structure of psychopathology in younger samples^14,16^ have relied on structured clinical interviews to measure symptoms of MDD, generalized anxiety disorder, multiple types of phobias, and panic disorder for internalizing psychopathology, and symptoms of alcohol use disorder, drug use disorder, conduct disorder, and antisocial personality disorders for externalizing psychopathology. Yet conducting structured clinical interviews is not feasible in large population-based data sets with multiple project aims. Thus, we relied on the available self-reported measures and included some constructs that capture dimensional psychopathology rather than psychiatric symptoms per se (e.g., impulsivity). These limitations are especially pronounced in the HRS measures of externalizing psychopathology, likely because behaviors like aggression and rule-breaking are less among older adults. Antisocial behavior in childhood further places individuals at risk of early mortality or long-term incarceration^88^, suggesting that individuals with the highest levels of externalizing behavior may not be represented in the HRS. Nevertheless, externalizing disorders such as ADHD and substance use disorder are still common: between 3% and 4% of adults aged 55–85 meet the criteria for ADHD^89^ and 3.8% of adults over aged 55 meet the criteria for substance use disorder^6^. The non-significant associations between polygenic risk and externalizing behaviors in the HRS may be owing to limited measures (e.g., impulsivity, trait anger, state anger, number of drinks per day) that do not adequately capture the complexity of externalizing behaviors in this age group. Although the HRS is a large population-based study, future studies are needed to determine whether the factor structure and genetic associations reported in the current study are generalizable to the broader population of older adults or reflect artifacts of the limited phenotypic measures available in the HRS.

Second, the GWAS summary statistics that we used to construct PGSs did not exclusively focus on older adults. Although maximizing statistical power through increasing sample size is a key consideration in GWAS, PGSs constructed from GWAS in younger samples may not generalize to older adults. This is particularly relevant considering the negative association we observed between impulsivity in the current sample and the PGS of antisocial behavior, constructed from a GWAS of adolescents and young-to-middle age adults^47^. GWAS of psychiatric outcomes in pediatric cohorts^83,90^ are beginning to show that genetic risk alleles may vary by developmental stage. As the availability of genomic data increases, future research should consider age-stratified GWAS.

Third, our analyses only focused on a subset of the population: older US adults of European ancestry. Though the focus on older adults is a critical addition to research on the meta-structure of psychiatric disorders in adulthood, psychiatric genetics, and human genetics studies overall, are overwhelmingly Eurocentric^91^—a trend that reduces generalizability of all genetic work and is likely to exacerbate health disparities^92^. We did not include participants of African ancestry in the current study because the available GWAS were conducted in European samples and, thus, would not be comparable for methodological rather than substantive reasons.

## Conclusion

Using multiple genome-wide PGSs for psychiatric outcomes, validated phenotypic measures, and novel analytic techniques in a relatively large, population-based sample of older adults, we showed that a single general factor of psychopathology best explained the phenotypic meta-structure of psychopathology in older adults in the HRS. Moreover, although PGSs were non-specific in their associations with internalizing and externalizing outcomes, latent PGSs that aggregated genetic effects across several disorders explained more transdiagnostic variation than any individual PGS alone. These results inform a changing conceptualization of psychiatric diagnoses and their genetic etiology—from disorder-specific to transdiagnostic and dimensional.

## Supplementary information

Supplemental Material

## References

[1] Schaefer JD (2017). Enduring mental health: prevalence and prediction. J. Abnorm. Psychol..

[2] Scott KM (2016). Association of mental disorders with subsequent chronic physical conditions: world mental health surveys from 17 countries. JAMA Psychiatry.

[3] Steptoe A, Deaton A, Stone AA (2015). Subjective wellbeing, health, and ageing. Lancet.

[4] Mokdad AH (2018). The state of US health, 1990-2016: burden of diseases, injuries, and risk factors among US states. JAMA.

[5] Schulz R (2000). Association between depression and mortality in older adults: the Cardiovascular Health Study. Arch. Intern. Med..

[6] Reynolds K, Pietrzak RH, El-Gabalawy R, Mackenzie CS, Sareen J (2015). Prevalence of psychiatric disorders in U.S. older adults: findings from a nationally representative survey. World Psychiatry.

[7] Mather, M., Jacobsen, L. A. & Pollard, K. M. Aging in the United States. *Population Bulletin* 70, no. 2 (2015).

[8] Kessler RC, Chiu WT, Demler O, Walters EE (2005). Prevalence, severity, and comorbidity of 12-month DSM-IV disorders in the National Comorbidity Survey Replication. Arch. Gen. Psychiatry.

[9] Laborde‐Lahoz P (2015). Subsyndromal depression among older adults in the USA: prevalence, comorbidity, and risk for new-onset psychiatric disorders in late life. Int J. Geriatr. Psychiatry.

[10] Krueger RF, Markon KE (2006). Reinterpreting comorbidity: a model-based approach to understanding and classifying psychopathology. Annu Rev. Clin. Psychol..

[11] Clark LA, Watson D (1991). Tripartite model of anxiety and depression: psychometric evidence and taxonomic implications. J. Abnorm. Psychol..

[12] Krueger RF (2002). Etiologic connections among substance dependence, antisocial behavior and personality: modeling the externalizing spectrum. J. Abnorm Psychol..

[13] Lahey BB, Krueger RF, Rathouz PJ, Waldman ID, Zald DH (2017). A hierarchical causal taxonomy of psychopathology across the life span. Psychol. Bull..

[14] Neumann A (2016). Single nucleotide polymorphism heritability of a general psychopathology factor in children. J. Am. Acad. Child Adolesc. Psychiatry.

[15] Schuster J-P, Hoertel N, Le Strat Y, Manetti A, Limosin F (2013). Personality disorders in older adults: findings from the national epidemiologic survey on alcohol and related conditions. Am. J. Geriatr. Psychiatry.

[16] Kendler KS, Prescott CA, Myers J, Neale MC (2003). The structure of genetic and environmental risk factors for common psychiatric and substance use disorders in men and women. Arch. Gen. Psychiatry.

[17] Kendler KS (2011). The structure of genetic and environmental risk factors for syndromal and subsyndromal common DSM-IV axis I and all axis II disorders. Am. J. Psychiatry.

[18] Cho SB (2017). Using patterns of genetic association to elucidate shared genetic etiologies across psychiatric disorders. Behav. Genet..

[19] Pettersson E, Larsson H, Lichtenstein P (2016). Common psychiatric disorders share the same genetic origin: a multivariate sibling study of the Swedish population. Mol. Psychiatry.

[20] Bulik-Sullivan B (2015). An atlas of genetic correlations across human diseases and traits. Nat. Genet..

[21] Lee PH (2019). Genomic relationships, novel loci, and pleiotropic mechanisms across eight psychiatric disorders. Cell.

[22] Boyle EA, Li YI, Pritchard JK (2017). An expanded view of complex traits: from polygenic to omnigenic. Cell.

[23] Martin J, Taylor MJ, Lichtenstein P (2018). Assessing the evidence for shared genetic risks across psychiatric disorders and traits. Psychol. Med..

[24] Choi SW, Mak TS-H, O’Reilly PF (2020). Tutorial: a guide to performing polygenic risk score analyses [no. 9]. Nat. Protoc..

[25] Anderson JS, Shade J, DiBlasi E, Shabalin AA, Docherty AR (2019). Polygenic risk scoring and prediction of mental health outcomes. Curr. Opin. Psychol..

[26] Docherty AR (2018). Polygenic prediction of the phenome, across ancestry, in emerging adulthood. Psychol. Med..

[27] Krapohl E (2018). Multi-polygenic score approach to trait prediction. Mol. Psychiatry.

[28] Grotzinger AD (2019). Genomic structural equation modelling provides insights into the multivariate genetic architecture of complex traits [no. 5]. Nat. Hum. Behav..

[29] Turley P (2018). Multi-trait analysis of genome-wide association summary statistics using MTAG. Nat. Genet..

[30] Sonnega A (2014). Cohort profile: the Health and Retirement Study (HRS). Int J. Epidemiol..

[31] Smith, J., Ryan, L. H., FIsher, G. G., Sonnega, A. & Weir, D. R. *HRS Psychosocial and Lifestyle Questionnaire 2006–2016*. Survey Research Center, Institute for Social Research, University of Michigan. Retrieved from https://hrs.isr.umich.edu/documentation/user-guides (2017).

[32] Domingue BW (2017). Mortality selection in a genetic sample and implications for association studies. Int J. Epidemiol..

[33] Health and Retirement Study. *([RAND HRS 2010 and 2012 Fat Files]) public use dataset.* Produced and distributed by the University of Michigan with funding from the National Institute on Aging (grant number NIA U01AG009740) (2018).

[34] Watson, D. & Clark, L. A. *The PANAS-X: manual for the positive and negative affect schedule – expanded form.* University of Iowa (1994).

[35] Beck AT, Epstein N, Brown G, Steer RA (1988). An inventory for measuring clinical anxiety: psychometric properties. J. Consult. Clin. Psychol..

[36] Radloff LS (1977). The CES-D scale a self-report depression scale for research in the general population. Appl Psychol. Meas..

[37] Tellegen, A. *Multidimensional personality questionnaire manual*. University of Minnesota Press (1982).

[38] Forgays DK, Spielberger CD, Ottaway SA, Forgays DG (1998). Factor structure of the state-trait anger expression inventory for middle-aged men and women. Assessment.

[39] Kotov R (2017). The hierarchical taxonomy of psychopathology (HiTOP): a dimensional alternative to traditional nosologies. J. Abnorm. Psychol..

[40] Sanislow CA (2010). Developing constructs for psychopathology research: research domain criteria. J. Abnorm. Psychol..

[41] Okbay A (2016). Genetic variants associated with subjective well-being, depressive symptoms, and neuroticism identified through genome-wide analyses. Nat. Genet..

[42] Otowa T (2016). Meta-analysis of genome-wide association studies of anxiety disorders. Mol. Psychiatry.

[43] Wray NR (2018). Genome-wide association analyses identify 44 risk variants and refine the genetic architecture of major depression. Nat. Genet..

[44] Walters RK (2018). Transancestral GWAS of alcohol dependence reveals common genetic underpinnings with psychiatric disorders. Nat. Neurosci..

[45] Demontis D (2019). Discovery of the first genome-wide significant risk loci For ADHD. Nat. Genet..

[46] Pasman JA (2018). GWAS of lifetime cannabis use reveals new risk loci, genetic overlap with psychiatric traits, and a causal effect of schizophrenia liability [no. 9]. Nat. Neurosci..

[47] Tielbeek JJ (2017). Genome-wide association studies of a broad spectrum of antisocial behavior. JAMA Psychiatry.

[48] Ware, E. B., Schmitz, L. L., Gard, A. M. & Faul J. *HRS Polygenic Scores – Release 3*. Survey Research Center, Institute for Social Research, University of Michigan (2018).

[49] Wood AR (2014). Defining the role of common variation in the genomic and biological architecture of adult human height. Nat. Genet..

[50] R. Core Team. *R: A Language and Environment for Statistical Computing*, version 3.6.3. R Foundation for Statistical Computing. Retrieved from: https://www.R-project.org/ (2020).

[51] Kline, R. B. *Principles and Practice of Structural Equation Modeling*. (Guilford Press, 1998).

[52] Schreiber JB, Nora A, Stage FK, Barlow EA, King J (2006). Reporting structural equation modeling and confirmatory factor analysis results: a review. J. Educ. Res..

[53] Bentler PM, Bonett DG (1980). Significance tests and goodness of fit in the analysis of covariance structures. Psychol. Bull..

[54] Cheung GW, Rensvold RB (2002). Evaluating goodness-of-fit indexes for testing measurement invariance. Struct. Equ. Modeling.

[55] Chen FF (2007). Sensitivity of goodness of fit indexes to lack of measurement invariance. Struct. Equ. Modeling.

[56] Rosseel Y (2012). lavaan: an R package for structural equation modeling. J. Stat. Softw..

[57] Graham JW (2009). Missing data analysis: making it work in the real world. Annu. Rev. Psychol..

[58] Jorgensen, T. D., Pornprasertmanit, S., Schoemann, A. M. & Rosseel, Y. *SemTools: Useful Tools for Structural Equation Modeling*., version 0.5-2. Retrieved from https://CRAN.R-project.org/package=semTools (2019).

[59] Park S, Smith J, Dunkle RE, Ingersoll-Dayton B, Antonucci TC (2019). Health and social–physical environment profiles among older adults living alone: associations with depressive symptoms. J. Gerontol. Ser. B.

[60] Ferraro KF (1980). Self-ratings of health among the old and the old-old. J. Health Soc. Behav..

[61] Ryan LH, Smith J, Antonucci TC, Jackson JS (2012). Cohort differences in the availability of informal caregivers: are the boomers at risk?. Gerontologist.

[62] Vandenberg RJ, Lance CE (2000). A review and synthesis of the measurement invariance literature: suggestions, practices, and recommendations for organizational research. Organ Res. Methods.

[63] Faul F, Erdfelder E, Lang A-G, Buchner A (2007). G*Power3: a flexible statistical power analysis program for the social, behavioral, and biomedical science. Behav. Res. Methods.

[64] Ben-Schacar, M. S. et al. *Effectsize: Indices of Effect Size and Standardized Parameters*., version 0.4.3. Retrieved from https://CRAN.R-project.org/package=effectsize (2021).

[65] Lahey BB (2009). Public health significance of neuroticism. Am. Psychol..

[66] Khan AA, Jacobson KC, Gardner CO, Prescott CA, Kendler KS (2005). Personality and comorbidity of common psychiatric disorders. Br. J. Psychiatry.

[67] Markon KE, Krueger RF, Watson D (2005). Delineating the structure of normal and abnormal personality: an integrative hierarchical approach. J. Pers. Soc. Psychol..

[68] Tackett JL (2012). The hierarchical structure of childhood personality in five countries: continuity from early childhood to early adolescence. J. Pers..

[69] Rodriguez-Seijas C, Eaton NR, Krueger RF (2015). How transdiagnostic factors of personality and psychopathology can inform clinical assessment and intervention. J. Pers. Assess..

[70] Barlow DH, Allen LB, Choate ML (2004). Toward a unified treatment for emotional disorders. Behav. Ther..

[71] Barlow, D. H., et al. *Unified Protocol for Transdiagnostic Treatment of Emotional Disorders: Therapist Guide*. Oxford University Press (2017).

[72] Ruderfer DM (2018). Genomic dissection of bipolar disorder and schizophrenia, including 28 subphenotypes. Cell.

[73] Edwards AC (2012). Genome-wide association study of comorbid depressive syndrome and alcohol dependence. Psychiatr. Genet..

[74] Traylor M, Markus H, Lewis CM (2015). Homogeneous case subgroups increase power in genetic association studies. Eur. J. Hum. Genet..

[75] Power RA (2017). Genome-wide association for major depression through age at onset stratification: major depressive disorder Working Group of the Psychiatric Genomics Consortium. Biol. Psychiatry.

[76] Lahey BB, Hulle CAV, Singh AL, Waldman ID, Rathouz PJ (2011). Higher-order genetic and environmental structure of prevalent forms of child and adolescent psychopathology. Arch. Gen. Psychiatry.

[77] Solovieff N, Cotsapas C, Lee PH, Purcell SM, Smoller JW (2013). Pleiotropy in complex traits: challenges and strategies. Nat. Rev. Genet..

[78] Maier RM, Visscher PM, Robinson MR, Wray NR (2018). Embracing polygenicity: a review of methods and tools for psychiatric genetics research. Psychol. Med..

[79] Hirschhorn JN, Daly MJ (2005). Genome-wide association studies for common diseases and complex traits. Nat. Rev. Genet..

[80] Malhotra D, Sebat J (2012). CNVs: harbingers of a rare variant revolution in psychiatric genetics. Cell.

[81] Dudbridge F (2013). Power and predictive accuracy of polygenic risk scores. PLOS Genet..

[82] Derringer J (2015). Genome-wide association study of behavioral disinhibition in a selected adolescent sample. Behav. Genet..

[83] Pappa I (2015). A genome‐wide approach to children’s aggressive behavior: the EAGLE consortium. Am. J. Med. Genet. B Neuropsychiatr. Genet..

[84] Mallard, T. T. et al. Multivariate GWAS of psychiatric disorders and their cardinal symptoms reveal two dimensions of cross-cutting genetic liabilities. *bioRxiv* https://www.biorxiv.org/content/10.1101/603134v2 (2020). 10.1016/j.xgen.2022.100140 PMC9264403 35812988

[85] Krueger RF, Eaton NR (2015). Transdiagnostic factors of mental disorders. World Psychiatry.

[86] First, M. B., Williams Janet, B. W., Spitzer, R. L. & Gibbon, M. *Structured clinical interview for DSM-IV axis I disorders*. New York State Psychiatric Institute (1995).

[87] Makol BA (2020). Integrating multiple informants’ reports: how conceptual and measurement models may address long-standing problems in clinical decision-making. Clin. Psychol. Sci..

[88] Shepherd JP, Shepherd I, Newcombe RG, Farrington D (2009). Impact of antisocial lifestyle on health: chronic disability and death by middle age. J. Public Health.

[89] Michielsen M (2012). Prevalence of attention-deficit hyperactivity disorder in older adults in the Netherlands. Br. J. Psychiatry.

[90] Middeldorp CM (2016). A genome-wide association meta-analysis of attention-deficit/hyperactivity disorder symptoms in population-based pediatric cohorts. J. Am. Acad. Child Adolesc. Psychiatry.

[91] Martin AR, Teferra S, Möller M, Hoal EG, Daly MJ (2018). The critical needs and challenges for genetic architecture studies in Africa. Curr. Opin. Genet. Dev..

[92] Martin AR (2019). Clinical use of current polygenic risk scores may exacerbate health disparities [no. 4]. Nat. Genet..

