# Supplementary material for: Phenotypic and genetic markers of psychopathology in a population-based sample of older adults
Source: Transl Psychiatry. 2021 Apr 24;11:239. doi: 10.1038/s41398-021-01354-2 (PMC8068727; doi:10.1038/s41398-021-01354-2)
Supplement: Supplementary file 1 — Supplemental Material [file 41398_2021_1354_MOESM1_ESM.docx]

**Supplemental Materials:**

**Phenotypic and Genetic Markers of Psychopathology in a Population-Based Sample of Older Adults**

Arianna M. Gard, PhD^1,2^, Erin B. Ware, PhD^2^, Luke W. Hyde, PhD^2,3,4^, Lauren Schmitz, PhD^5^, Jessica Faul, PhD^2^, & Colter Mitchell, PhD^2^

^1^Department of Psychology, University of Maryland, College Park

^2^Institute for Social Research, University of Michigan, Ann Arbor

^3^Department of Psychology, University of Michigan, Ann Arbor

^4^Center for Growth and Human Development, University of Michigan, Ann Arbor

^5^La Follette School of Public Affairs, University of Wisconsin

**Supplemental Methods**

**Genetic Ancestry Estimation**

SNPs used for PCA were selected by LD pruning from an initial pool consisting of all autosomal SNPs with a missing call rate < 5% and minor allele frequency (MAF) > 5%, and excluding any SNPs with a discordance between HapMap controls genotyped along with the study samples and those in the external HapMap data set. In addition, the 2q21 (LCT), HLA, 8p23, and 17q21.31 regions were excluded from the initial pool (1).

Additional information about the genetic quality-control procedures in the Health and Retirement Study can be found at https://hrsonline.isr.umich.edu/sitedocs/genetics/HRS2_qc_report_SEPT2013.pdf?_ga=2.59963114.6513449.1600955376-325044460.1531409717&_ga=2.59963114.6513449.1600955376-325044460.1531409717

**Supplemental Results**

**Measurement Invariance by Gender**

Women reported greater negative affect (t[5923] = 7.31, *p* < .001), anxiety (t[5928] = 3.77, *p* < .001) and depressive symptoms (t[5965] = 7.02, *p* < .001), and men reported greater state anger (t[5927] = 5.05, *p* < .001). Despite mean level gender differences, the one-factor model of general psychopathology fit well in both men and women (Figure 3a). Measurement invariance testing revealed metric invariance across genders: fixing the indicator loadings to be equivalent across groups did not significantly degrade model fit. Not surprisingly, given mean differences in the individual measures by gender, the model did not meet criteria for scalar measurement invariance (i.e., equivalent intercepts across groups), indicated by ΔCFI = .031 and ΔTLI = .015.

**Measurement Invariance by Age**

Middle age (51 – 64 years; *n* = 2,212), young-old (65 – 74, *n* = 2,366), and old-old (75 – 83, *n* = 1,425) HRS participants significantly differed in their mean levels of negative affect (F[2,5922] = 47.64, *p* < .001), trait anger (F[2,5935] = 44.72, *p* < .001), state anger (F[2,5926] = 22.003, *p* < .001), impulsivity (F[2,5918] = 3.04, *p* < .05), and depressive symptoms (F[2,5998] = 73.33, *p* < .001). Tukey post-hoc comparisons revealed that middle age participants reported greater negative affect, trait anger, state anger, and depressive symptoms than young-old participants (all *p*s < .01). Young-old participants, in turn, reported greater trait and state anger than old-old participants, but did not differ from old-old participants on negative affect or depressive symptoms. Lastly, although middle-age and young-old participants did not differ on self-reported impulsivity, old-old participants reported greater impulsivity than both younger age groups. Despite mean-level differences in phenotypic outcomes by age group, the one-factor model of general psychopathology fit well in all three age groups (Figure 3b). As in invariance testing by gender, metric (i.e., fixed loadings), but not scalar (i.e., fixed intercepts), invariance was established across age groups (Figure 3b).

Supplemental Table 1. Descriptive statistics and sources of phenotypic measures

| **Phenotypic Measures of Psychopathology** | | | | |  |
| --- | --- | --- | --- | --- | --- |
| **Construct** | **Mean (SD)** | **Min – Max** | **Reliability (α)** | **Number of Items** | **Source** |
| Negative Affect | 1.74 (.60) | 1 – 4.83 | .97 | 12 | Positive and Negative Affect Schedule – Expanded Form (2) |
| Anxiety Symptoms | 1.51 (.55) | 1 – 4 | .97 | 5 | Beck Anxiety Inventory (3) |
| Depressive Symptoms | 1.12 (1.76) | 0 – 8 | .98 | 8 | Center for Epidemiologic Studies Depression Scale (4) |
| Impulsivity | 2.69 (.83) | 1 – 6 | .97 | 6 | Multidimensional Personality Questionnaire (5) |
| Trait Anger | 2.21 (.69) | 1 – 4 | .96 | 4 | State-Trait Anger Expression Inventory (6) |
| State Anger | 1.49 (.49) | 1 – 4 | .97 | 7 | State-Trait Anger Expression Inventory (6) |
| Drinking Frequency* | .83 (1.41) | 0 – 24 | - | 1 | - |

Note. 5,921 > *N* > 6,001. All constructs except drinking frequency were measured as means of all items. *Participants were asked whether they currently consume alcoholic beverages and, if so, how many per day on average. A continuous measure was constructed, where non-drinkers were recoded as “0”.

Supplemental Figure 1. Non-zero cross-trait LD-score regression intercepts suggest cryptic relatedness among GWAS samples


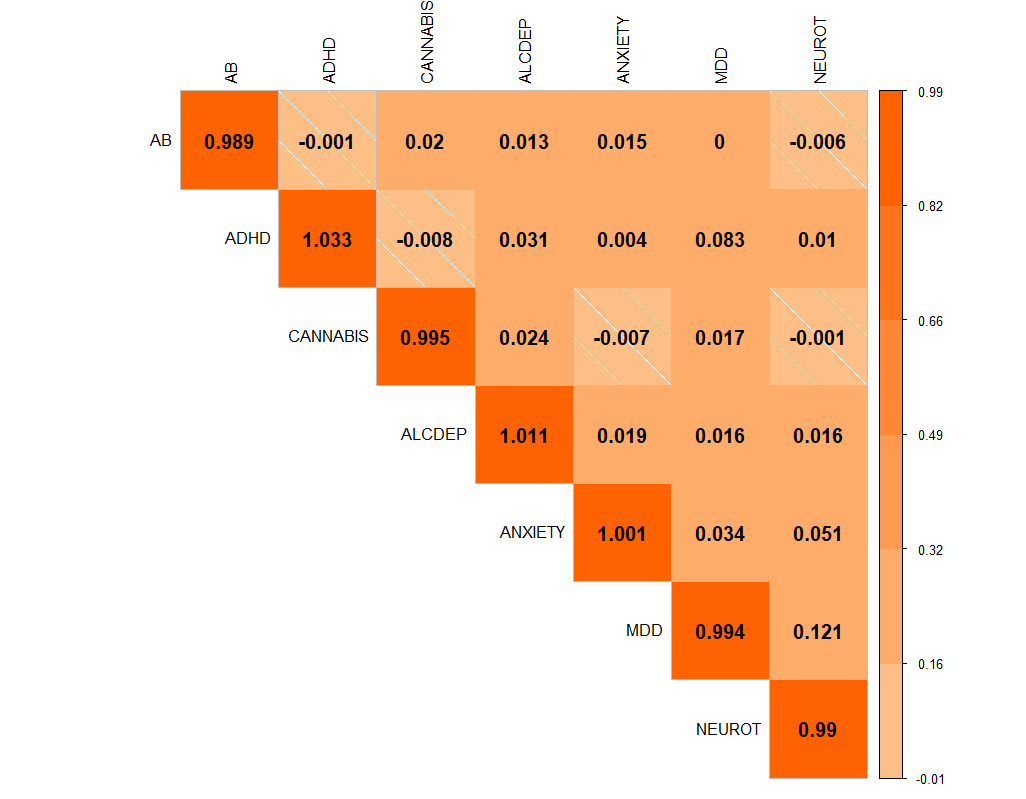


Supplemental Figure 2. Invariant indicator loadings of the general factor of psychopathology by age group and gender in the Health and Retirement Study


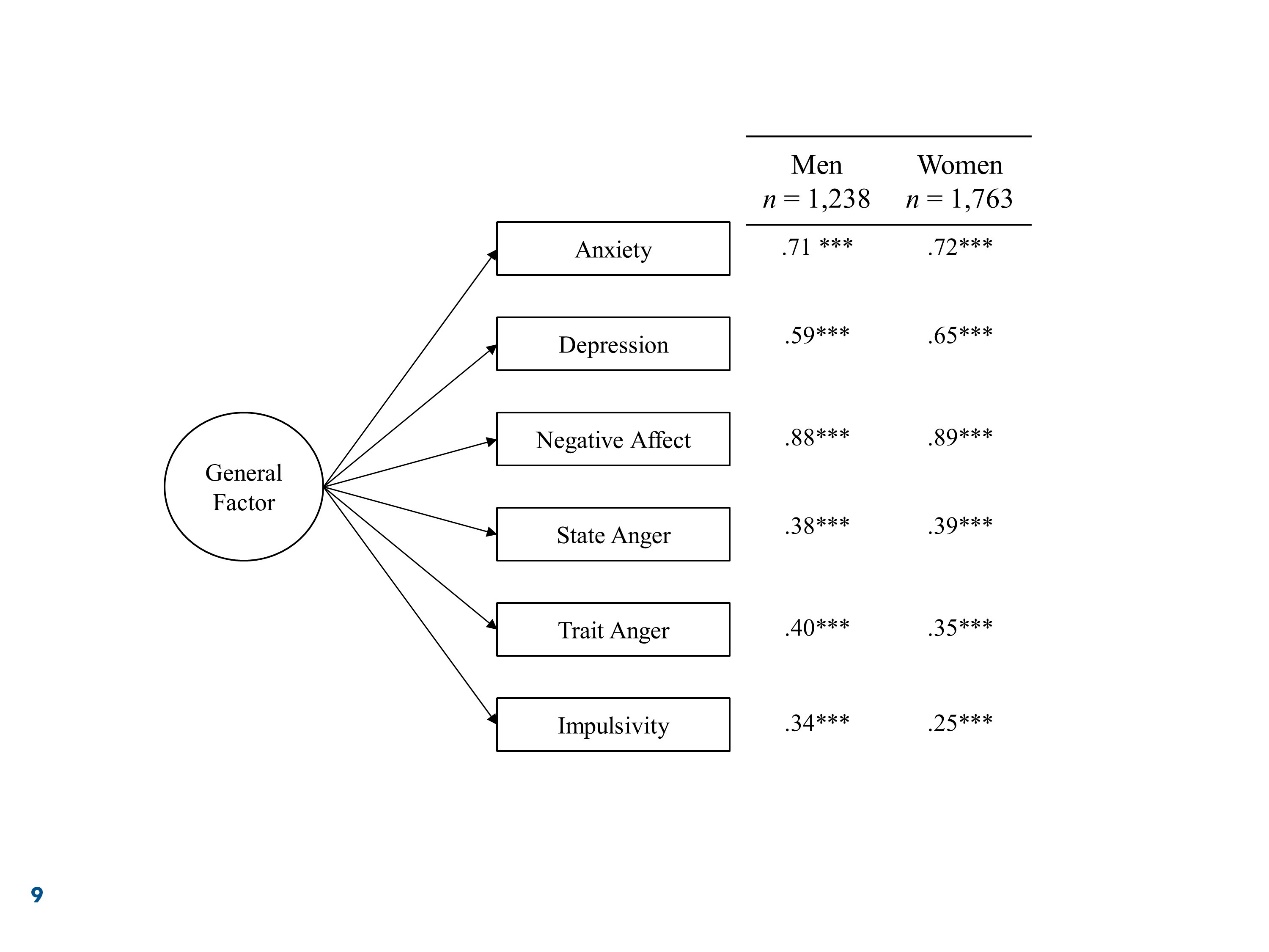


(A)


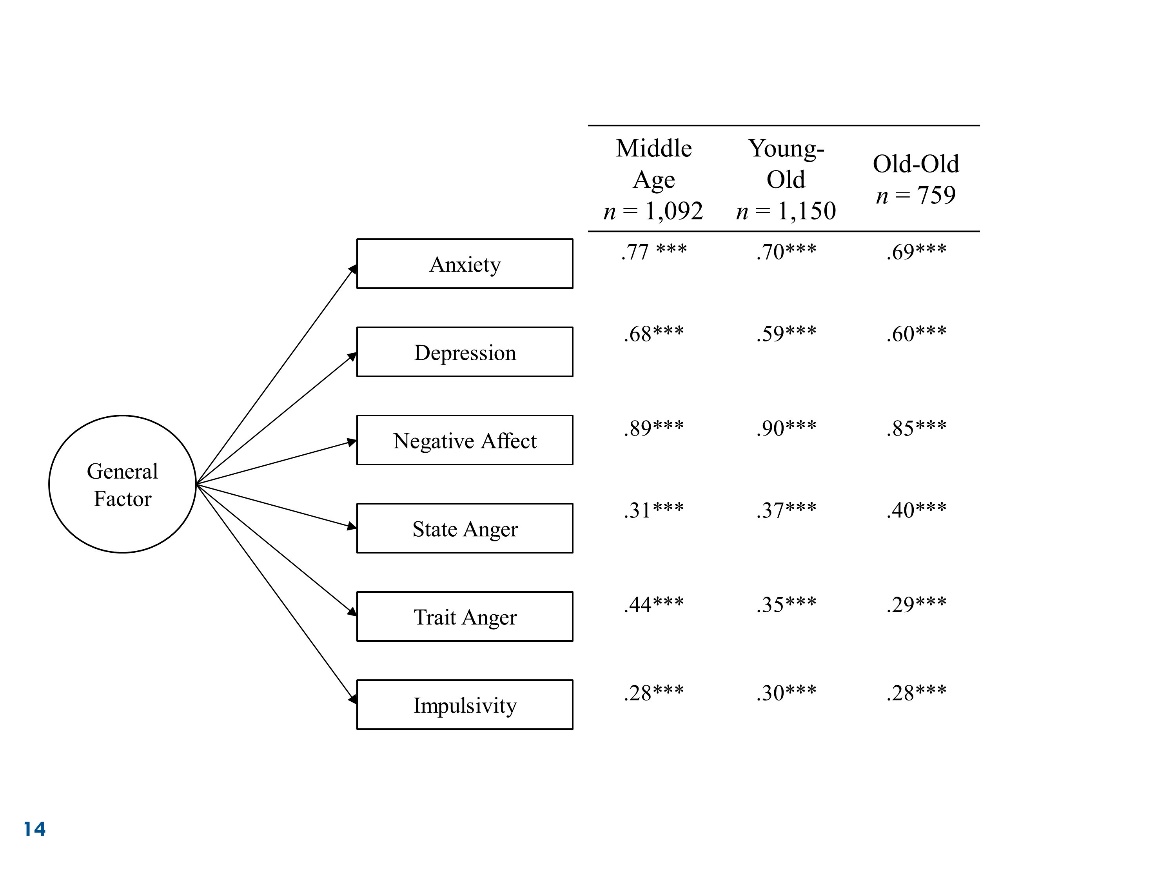


(B)

Note. (A) Confirmatory one-factor phenotypic models in the hold-out sample, split by gender (model fit: χ² (18) = 64.22, *p* < .001; CFI = .985; TLI = .975; RMSEA = .045, 90% CI [.033, .057]). Increasingly stringent measurement invariance testing revealed no significant change in model fit when loadings were fixed across groups (metric invariant model ΔCFI = .009, ΔRMSEA = 004), but a significant depreciation of model fit when loadings and intercepts were fixed across groups (scalar invariant model ΔCFI = .018, ΔRMSEA = .009). (B) Confirmatory one-factor phenotypic models in the hold-out sample, split by age group (model fit: χ² (27) = 82.77, *p* < .001; CFI = .983; TLI = .971; RMSEA = .049, 90% CI [.037, .061]). Increasingly stringent measurement invariance testing revealed no significant change in model fit when loadings were fixed across groups (metric invariant model ΔCFI = .009, ΔRMSEA = .003), but a significant depreciation of model fit when loadings and intercepts were fixed across groups (scalar invariant model ΔCFI = .031, ΔRMSEA = .015).

Supplemental Figure 3. Unstandardized factor loadings for Genomic SEM one-factor and two-factor models.


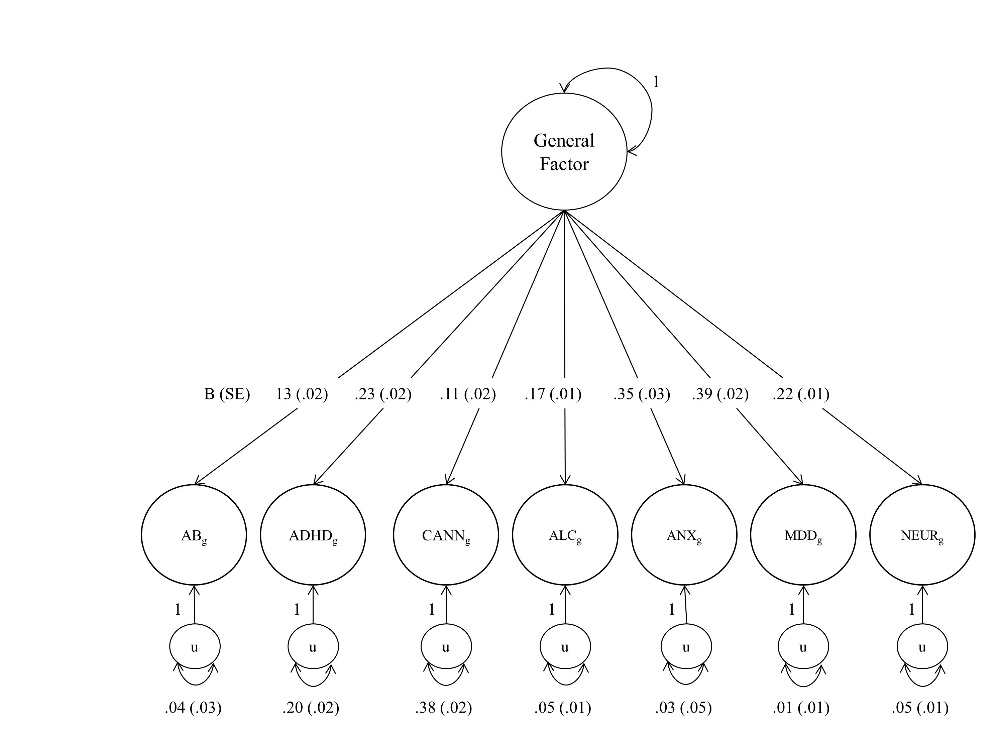


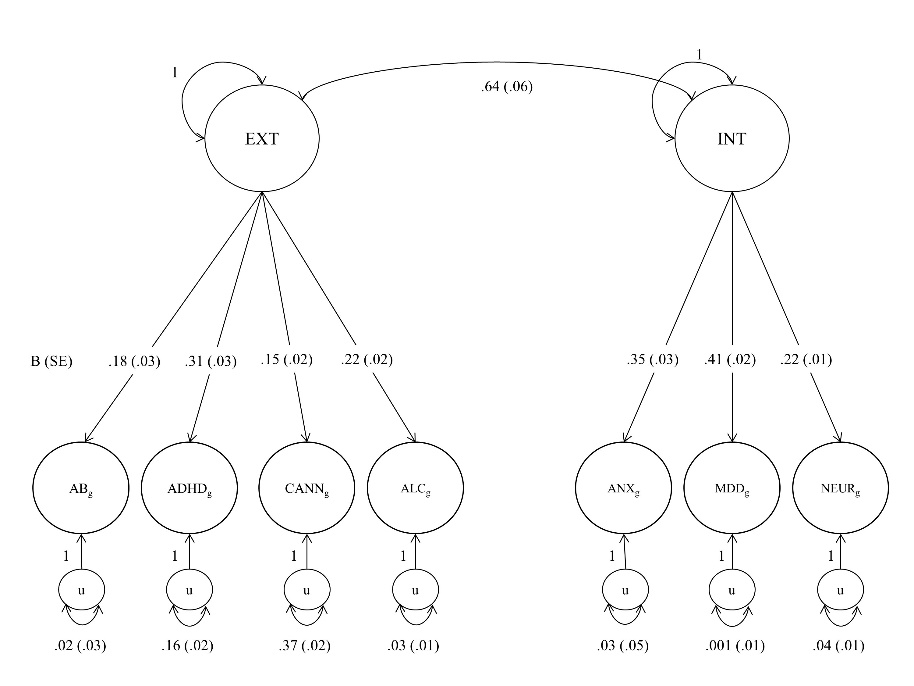


*Note.* Confirmatory factor analyses were conducted on the GWAS summary statistics in Table 1, using the Genomic SEM package in R Statistical Software (7). In both the one-factor and two-factor models, the residual variance of MDD was fixed to zero. Model fit comparisons between the one-factor model (χ² [14] = 76.762, p < .001, AIC = 104.762, CFI = .962, SRMR = .127) and two-factor model (χ² [13] = 46.072, p < .001, AIC = 76.072, CFI = .980, SRMR = .084) indicated superior model fit of the two-factor model (Δχ² = 30.69(1), p < .001, ΔCFI > .01, lower AIC). Single-nucleotide polymorphism effects were then integrated into the model to derive new SNP weights for construction of latent polygenic scores (see Supplemental Methods).

**References**

1. Ware EB, Schmitz LL, Gard AM, Faul J (2018): HRS Polygenic Scores – Release 3. Ann Arbor, MI: Survey Research Center, Institute for Social Research, University of Michigan.

2. Watson D, Clark LA (1994): The PANAS-X: Manual for the positive and negative affect schedule – expanded form. University of Iowa.

3. Beck AT, Epstein N, Brown G, Steer RA (1988): An inventory for measuring clinical anxiety: psychometric properties. *J Consult Clin Psychol* 56: 893.

4. Radloff LS (1977): The CES-D scale a self-report depression scale for research in the general population. *Appl Psychol Meas* 1: 385–401.

5. Tellegen A (1982): Brief Manual for the Multidimensional Personality Questionnaire. University of Minnesota.

6. Forgays DK, Spielberger CD, Ottaway SA, Forgays DG (1998): Factor Structure of the State-Trait Anger Expression Inventory for Middle-Aged Men and Women. *Assessment* 5: 141–155.

7. Grotzinger AD, Rhemtulla M, Vlaming R de, Ritchie SJ, Mallard TT, Hill WD, *et al.* (2019): Genomic structural equation modelling provides insights into the multivariate genetic architecture of complex traits [no. 5]. *Nat Hum Behav* 3: 513–525.
